# The feasibility of a self-regulation and mental imagery programme to enhance everyday functioning for people with Parkinson’s disease: study protocol

**DOI:** 10.1186/s40814-024-01578-1

**Published:** 2024-12-05

**Authors:** Christine Hill, Renai Pillay, Karen P. Y. Liu

**Affiliations:** 1 School of Health Sciences, Western Sydney University, Penrith, NSW Australia; 2 Department of Rehabilitation Sciences, The Hong Kong Polytechnic University, Kowloon, Hong Kong SAR; 3 Concord Hospital, Concord, NSW Australia

**Keywords:** Parkinson’s disease, Rehabilitation, Self-regulation, Mental imagery, Everyday activities

## Abstract

**Background:**

Less dopaminergic neurons in people with Parkinson’s disease result in a reliance on a slow and energy-intensive control, restricting their ability to complete routine everyday activities. Self-regulation takes an active learning approach to rehabilitation by enhancing the person’s self-awareness and encourages self-reflection to overcome problems. Mental imagery assists the person to focus attention on the requirements for the task, making it a goal-directed action and allowing for faster movements. There is a growing body of evidence to support the use of self-regulation and mental imagery in rehabilitation to maintain or improve the performance of everyday activities for people with a neurological condition.

**Methods:**

A prospective pilot study using a single-group, pre-test and posttest design will test the feasibility and acceptability of a self-regulation and mental imagery programme to enhance performance of everyday activities and motor and cognitive function in people with Parkinson’s disease. People who have a diagnosis of Parkinson’s disease with mild-to-moderate severity and intact attention, comprehension and short-term memory will be recruited. The participants will receive a 6-week programme with one therapist-led session, a home programme and a telephone call each week. The programme uses self-regulation through a step-based problem-solving process and mental imagery to assist in movement planning. Appropriate rehabilitation strategies are used as solutions to tackle problems experienced, impacting performance in everyday activities. The attendance rate will be recorded to indicate the feasibility. A questionnaire will be administered post-intervention to collect feedback on programme acceptability. Patient outcomes will include Barthel index, Lawton Instrumental Activities of Daily Living Scale, Canadian Occupational Performance Measure and Timed Up and Go test. Trail Making Test and Montreal Cognitive Assessment will be collected pre- and post-intervention.

**Discussion:**

The programme aims to combine the metacognitive strategies of self-regulation and mental imagery to enable individuals with Parkinson’s disease to improve the performance of everyday activities. If feasible, the programme has the potential to be further tested in a randomised controlled trial and benefit people with Parkinson’s disease by enhancing their performance required in independent community living.

**Trial registration:**

Australian New Zealand Clinical Trials Registry ACTRN12621000903886, Registered on 12 July 2021 — retrospectively registered.

**Supplementary Information:**

The online version contains supplementary material available at 10.1186/s40814-024-01578-1.

## Background

An estimated 6.1 million people worldwide were living with Parkinson’s disease (PD) in 2016 [1]. The majority are over the age of 55 years, living in the community and cared for at home [2]. PD is a neurodegenerative condition caused by the loss of dopaminergic neurons in the substantia nigra of the basal ganglia [3, 4]. The loss of dopaminergic neurons in this specific region of the brain results in an impairment to the supervisory attentional system (SAS) affecting a person’s mental flexibility and ability to focus their attention on movements [5]. It results in losing habitual control and a reliance on a slow and energy-intensive action when the person engages in movement required in everyday activities [6]. This results in characteristic motor symptoms of slow movements, poor initiation, rigidity, poor balance, poor coordination and poor execution of movements in people with PD as well as cognitive deficits such as memory problems, a greater reaction time and poor executive function [3, 7]. All these motor and cognitive problems affect a person with PD’s ability to participate in everyday activities [8].

To manage the symptoms of PD and improve the performance of everyday activities, medication such as a dopamine agonist is commonly used [9]. However, as the disease progresses, the medication effect wears off [10, 11]. For this reason, 40% of people diagnosed with PD would require non-pharmacological rehabilitation interventions beyond standard medication treatment [9, 10].

Effective rehabilitation used for people with PD includes exercise, equipment prescription, home modifications, positive coping strategies and fatigue management [3, 12]. We propose that these rehabilitation strategies can be classified into three main types focusing on the following: (1) modifying the ‘Task’, (2) adapting the ‘Environment’ and (3) seeking for ‘Assistance’ (‘TEA’). When modifying the ‘Task’, the individual can employ positive coping strategies and fatigue management. Examples are sitting down while performing the task, using precut meat or vegetables for cooking. Using equipment or having home modifications is examples of adapting the ‘Environment’. External cueing has been found to be useful to enhance movement initiation for people with PD [3, 13–15]. It has been shown to improve overall functioning for people with PD [13, 16]. External cueing is an attentional strategy that uses visual or auditory stimuli in the environment to promote movement [15]. Examples include using lines on floor to assist walking to reduce the attention required on spatial planning and using metronome to pace movement and assist with timing of steps [17, 18]. External cues take the role of a pace setter, reducing the attention required to intiate movement and allowing for attention to be used on other aspects of the movements, therefore compensating for the impaired SAS [19]. It can also assist the brain to switch to goal-directed motor control when a person experiences freezing symptoms [20]. We propose the use of external cueing as a form of adapting the ‘Environment’. When both modifying the ‘Task’ and adapting the ‘Environment’ do not work in enhancing everyday activities performance, individual may seek ‘Assistance’ from others. Except for the use of external cueing, these rehabilitation strategies adopt a compensatory or health promotion approach and do not directly work with the deficits in SAS which cause the reduced everyday activity performance described earlier. Self-regulation and mental imagery are proposed to focus attention and provide goal-directed control on movements that may work on the deficits in SAS.

Self-regulation takes an active learning approach to rehabilitation by enhancing the person’s self-awareness and encourages self-reflection to overcome problems [21, 22]. Extensive research has shown that self-regulation enhances everyday activity performance for individuals who have had a stroke or traumatic brain injury [21–23]. Self-regulation uses active awareness of difficulties to enable the person to problem solve and implement solutions [24]. A recent study by Tinaz and Elfil [25] examined the neural correlation of self-regulation, and the result supported the use of self-regulation for people with mild PD. By a person focusing their attention on the task they are having difficulties with, they are able to use a goal-directed approach to complete the task [25]. There is unclear evidence focusing on the benefit of incorporating self-regulation with other rehabilitation techniques for people with PD [26]. With the evidence on the benefit of self-regulation on people with stroke [21, 22, 24], we proposed that self-regulation will do the same on people with PD by enabling them to reflect on their capacity, bring to a conscious level their difficulties and focus their attention on their performance of everyday activities using solutions they generate in order to promote the use of the goal directed brain pathways and overcome the limitations as a result of the deficit in the SAS [25, 27].

Mental imagery involves imagining every detail of the actions required to perform a movement or an activity without the overt physical movement [28]. It is a low-cost and low-risk intervention that evokes similar neural responses to physically doing a task allowing for multiple repetitions without the physical fatigue [29]. Through the process, mental imagery assists the person to focus attention on the requirements for the activity. It can be regarded as an attentional strategy that will allow people with PD to plan and prepare for the sequence of moves required for the task before doing them and include any new method or equipment gained during rehabilitation [30]. We propose that mental imagery helps the person to overcome the impairment to the SAS and making goal-directed action during the movements [20]. It may, therefore, assist with the common PD symptoms limiting activity performance such as freezing and bradykinesia [6].

Using the strategies on modifying the ‘Task’ and ‘Environment’ and getting ‘Assistance’ (‘TEA’) as potential solutions to solve problems encountered in the performance of everyday activities, it is proposed that self-regulation and mental imagery are useful to enhance people with PD to plan, monitor and control their movements in a goal-directed manner, possibly compensating for the impairment in the SAS caused by the loss of dopaminergic neurons.

### Study aims and hypothesis

This study aims to investigate the feasibility and acceptability of the newly developed ‘Self-Regulation and Mental Imagery for Parkinson’s disease’ (SReMI-PD) programme, aiming at enhancing the performance of everyday activities, motor function and cognitive function in people with PD.

This study hypothesises that goal-directed attention and self-regulation built in the SReMI-PD programme will be feasible and acceptable to enhance performance of everyday activities for people with PD.

## Methods

### Trial design and setting

This pilot experimental study will be conducted using a single-group, pre-test and post-intervention design evaluating the feasibility, acceptability and potential outcomes of the SReMI-PD programme. The protocol follows the Standard Protocol Items: Recommendations for Intervention Trials 2013 statement [31]. The SReMI-PD programme will be conducted at the outpatient Parkinson’s Clinic, Concord Repatriation General Hospital, Sydney, Australia.

### Participants

People who have a diagnosis of PD will be included if they fulfil the following inclusion criteria: (1) aged 18 and above; (2) diagnosed with PD by an neurologist; (3) have mild-to-moderate severity of PD with scores of 2, 2.5 and 3 on Hoehn and Yahr scale [32]; and (4) have been screened for intact attention, comprehension and short-term memory assessed using the Montreal Cognitive Assessment [33]. Participants with language barriers that may cause difficulties participating in the intervention will be excluded from the study. The carers of people with PD will be able to attend and observe the intervention.

### Recruitment

**Fig. 1:**
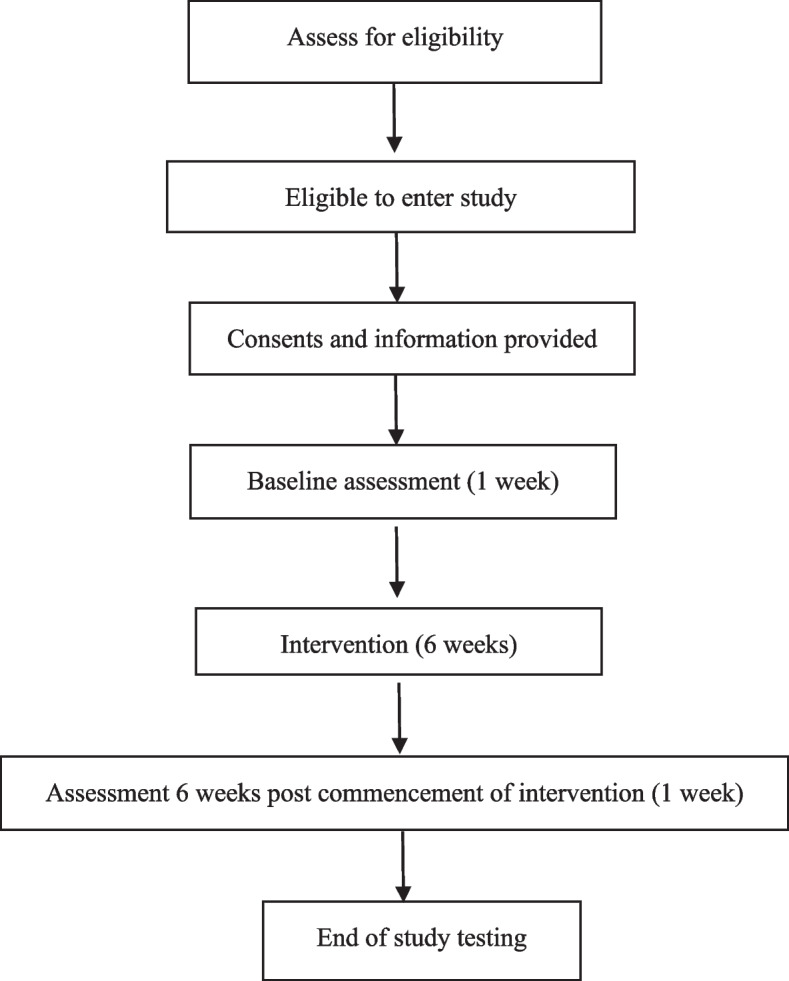
Consort diagram of the study

Participants will be recruited through promotional flyers being handed out at the Parkinson’s Clinic at Concord Repatriation General Hospital. The occupational therapists and physiotherapist at the clinic will also identify and invite people who meet the selection criteria (Fig. 1). The potential participants will be provided with the participant information sheet and contacted by one of the researchers. The participant information sheet explains the study, including its aims, what participation involves, the voluntary nature of participation and confidentially. Individuals who are interested will also be able to contact the researchers for further information. Researchers who are not involved in data collection will screen the individuals to ensure they meet all the selection criteria for the study. Persons fulfilling the selection criteria will receive written and oral information about the study. This will include general information including in the participant information sheet, such as voluntary nature of participation, rights to withdrawal and confidentiality, information about the pre-intervention assessments required and information regarding the intervention. The individuals can decide on their participation immediately. If they wish to consider participating, they are free to take the participant information sheet and contact the researchers anytime they wish to participate. Informed written consent will be collected prior to the pre-intervention assessments being completed. All participants are free to withdraw from the study at any time, and this will be explained to participants.

### Intervention

#### Development of the programme

The SReMI-PD programme was developed based on a review of theory and scientific evidence on people with stroke as well as two consumer co-design workshops. Previous studies reviewed the benefits of using self-regulation and mental imagery in promoting relearning and generalisation of skills learnt for people with stroke and traumatic brain injuries [21–24, 34]. The two strategies are proposed to assist people with PD in focusing attention on task requirements. The two consumer co-design workshops included five health professionals working with people with PD and three people with PD and their carers. The workshops gathered clinical experience of these health professionals and the needs and expectations of persons living with PD and their carers in the intervention programme design.

#### Duration and specific content of the programme

The SReMI-PD programme runs for 6 weeks with a 45-min face-to-face group session, a home programme and a follow-up phone call. The weekly face-to-face group sessions will involve between two to four members per group.

At the start of the programme, the participants will identify a list of everyday activities that they feel challenging. The programme will begin with 2 weeks of learning and applying self-regulation through a step-based problem-solving process. This will include choosing a task from the list of everyday activities identified, breaking the task down into steps, identifying steps that are challenging or difficult, identifying what the specific problems they are experiencing and brainstorming solutions. Throughout the programme, appropriate rehabilitation strategies will be suggested and used to build the participants knowledge of possible solutions. They will include modifying the ‘Task’ and ‘Environment’ and getting ‘Assistance’ (‘TEA’). The acronym ‘TEA’ for ‘Task, Environment, Assistance’ will be taught to assist participants with coming up with solutions. External cueing will be introduced as part of the strategies for adapting the ‘Environment’ as a potential solution. Participants will be given the opportunity to try the various solution brainstormed during the session and then encouraged to implement the chosen solutions at home. The third and fourth sessions will introduce mental imagery into the self-regulation process as a method of practicing the task. Mental imagery will assist the participants to plan their movements during a task performance. The final two sessions will consolidate the use of self-regulation and mental imagery and the solutions using the ‘TEA’ concept with various everyday activities while considering current difficulties and potential future difficulties.

The home programme includes the activities practiced during the face-to-face session that week. It aims at encouraging participants to implement the strategies and solutions at home. To monitor the adherence to the home programme, a telephone phone call will be given to the participants to check on any issues or extra support required.

#### Training of the intervention providers

The intervention will be facilitated by an occupational therapy fourth-year honours student and two registered occupational therapists. Throughout the intervention period, the honours student will receive supervision to approve the programme plan for the week and to clarify any issues related to the programme application in clinical practice.

To ensure uniformity and standardisation of the programme, a comprehensive programme plan describing the procedures, content of each session and the home programme has been developed. Before the programme and each week, the therapists and occupational therapy student involved in the intervention will discuss the plan.

After each face-to-face group session, the therapists and occupational therapy students will discuss and clarify issues related to the programme and the response of the participants. Modifications to the content may be made according to the participants’ response each week.

### Data collection

#### Demographic data

Demographic data will be collected at the baseline assessment, prior to beginning the intervention. The information collected includes their age, sex, years since diagnosis of PD and current medication. The Hoehn and Yahr scale [32] and the Unified Parkinson’s Disease Rating Scale [35] will be completed before the intervention.

#### Primary feasibility outcomes

##### Feasibility

Participant’s attendance in each face-to-face group session, a record of their participation in the home programme and the follow-up phone call will be collected to determine the feasibility of the programme.

##### Acceptability

At the end of the 6-week programme, participants will be asked to partake in a structured interview where questions from a developed form will be asked around the acceptability of the intervention programme. The questions will reflect themes of programme acceptability as suggested by Huxley and Mohamad [36] and Sidani and Epstein [37]. They include four attributes (perceived effectiveness, relevancy, convenience and clarity) and four features (face-to-face sessions, self-administered home sessions, accompanying resources and overall programme). These questions will be examined through a 4-point Likert scale where ‘4’ represents ‘completely agree’ and ‘1’ represents ‘completely disagree’.

##### Secondary patient-centred outcomes

Participants’ daily task performances, motor and cognitive function will be assessed at baseline and 6-week post-commencement of intervention using the Barthel index, the Lawton Instrumental Activities of Daily Living Scale, the Canadian Occupational Performance Measure, the Timed Up and Go test and the Trail Making Test. These outcome measures will be conducted by an independent occupational therapist who has no personal or professional relation to the research or the intervention programme.

The Barthel index is a standardised test to evaluate an individual with a neuromuscular or musculoskeletal disorder’s independence in caring for themselves [38]. It covers a range of self-care tasks including feeding, bathing, grooming, dressing, toileting, transfers and mobility [38]. The Lawton Instrumental Activities of Daily Living was created to assess individuals’ everyday functional competence in the community including skills such as using the telephone, shopping, food preparation, housekeeping, laundry, transportation, medication management and financial management [39]. Together with the Barthel index, this assessment will show the participant’s overall everyday functioning and evaluate any changes as a result of the intervention.

The Canadian Occupational Performance Measure is a standardised client-centred outcome measure that assessed an individual’s self-perception of performance in everyday living [40]. It is administered in a semi-structured interview format where issues related to performance of everyday activities are identified; rated in importance and ability to perform, and finally their satisfaction with their performance; and are sensitive to change over time [41]. This instrument focuses on the participants’ perceptions of their functioning and improvements which is valuable in identifying the areas to focus on in the intervention programme as well as determine their perceived improvements as a result of the intervention programme.

The Timed Up and Go test is a standardised evaluation that looks at the time it takes for an individual to rise from an arm chair, walk to a line 3 m away and turn and return to sit on the chair again [42]. The score an individual receives can be compared to normative values as well as be used as an outcome measure to evaluate changes [43]. The Timed Up and Go test is able to quantify a person’s functional mobility and ability to mobilise independently outside safely [42].

The Trail Making Test assess a person’s executive functioning, visual scanning, attention, mental flexibility and motor functioning and is considered a sensitive test to detect different kinds of brain damage such as in Parkinson’s disease [44]. The Trial Making Test is beneficial in showing cognitive changes influenced by the strategies learnt during the intervention programme.

### Sample size

In this pilot study, a sample of 12 participants was chosen. The sample size is selected because of the rationale about feasibility, precision about the mean and variance and regulatory considerations discussed by Julious [45]. When there is no background information on which to select a sample size, 12 is used because statistical properties become stable [45]. If a participant drops out of the study, a new participant will be recruited to ensure the study concludes with a sample of 12 participants.

### Participant timeline

Enrolment of participants will begin once ethics approval is received. The study will be run between March 2021 and August 2021. During this period, 12 participants will be recruited and enrolled in the programme. At the time of submission of this study protocol, recruitment to the study is ongoing.

**Fig. 2:**
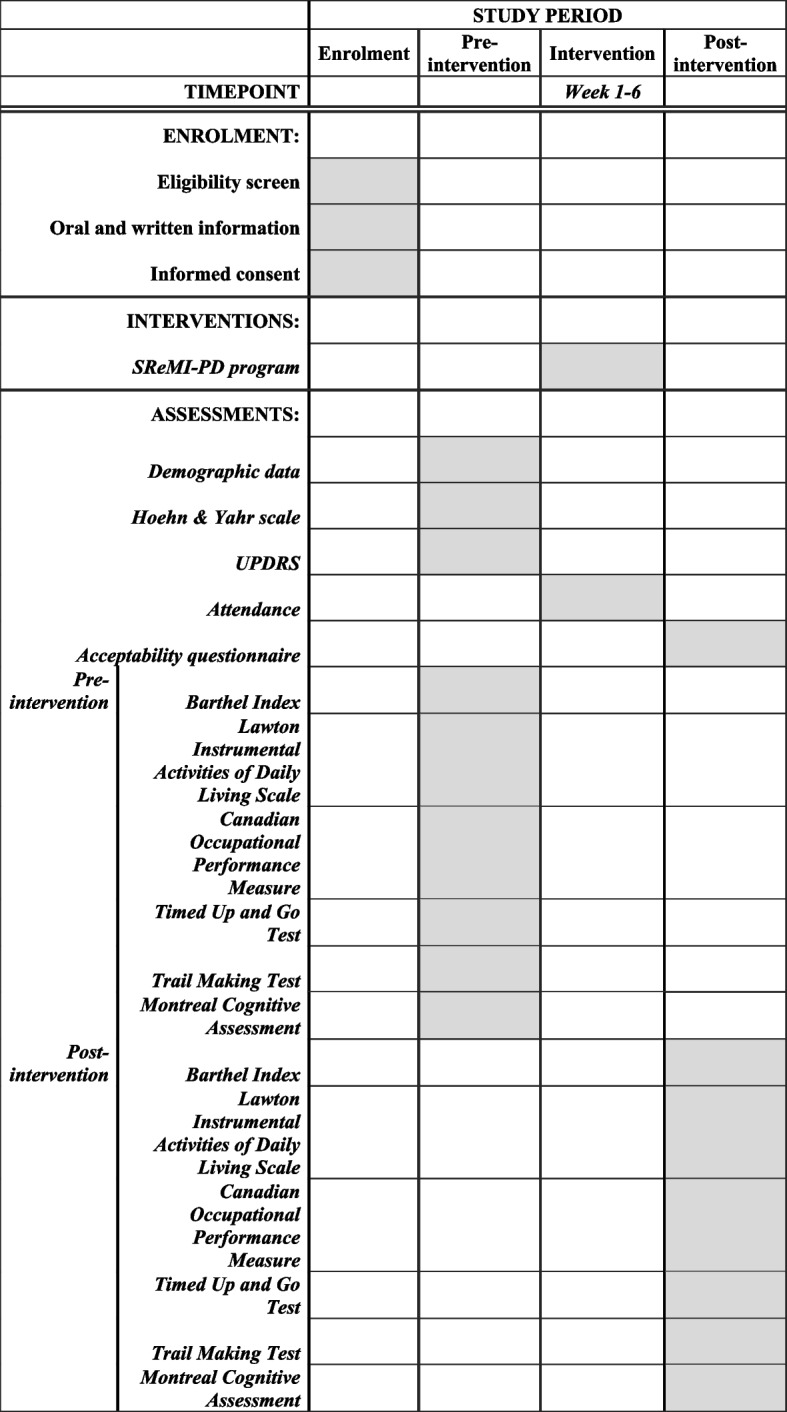
Standard Protocol Items: Recommendations for Interventional Trials (SPIRIT) figure of enrolment, interventions and assessments

Each participant will have their demographic data, the Hoehn and Yahr scale and pre-intervention assessment conducted after enrolment (Fig. 2; Additional File 1). The acceptability questionnaire and the post-intervention assessment will be conducted on the completion of the intervention programme. Participants’ attendance to the intervention programme will be recorded throughout the intervention programme.

### Data analysis

Descriptive statistics will be used to report all data collected. The number of persons recruited and the inclusion process will be presented in a CONSORT flow diagram. The attendance as represented by the retention rate and adherence to the intervention programme will be presented based on frequencies and percentages. The information will indicate the feasibility of the programme.

The results of the acceptability questionnaire will be combined, analysed categorically and reported to assess the programme acceptability. The additional comments from the participant will be reported.

The results from the assessments conducted pre- and post-intervention will be reported using the medians and interquartile ranges. Using the nonparametric Wilcoxon signed-rank tests, the differences between the pre- and post-intervention results will be analysed. All analyses will use the Statistical Package for Social Sciences version 25.0.

## Discussion

This current study will pilot-test the newly developed SReMI-PD programme and assess the feasibility and acceptability. This programme aims to combine the metacognitive strategies of self-regulation and mental imagery to enable individuals with PD to improve their performance of everyday activities. If the study’s results suggest that this programme is feasible with at least 80% of attendance and acceptable with medium ratings of 3 or above (agree and completely agree) in all areas as well as indicates positive outcomes, the intention is to test the outcomes in a randomised controlled trial in the future. Using attendance to evaluate the feasibility of the study may not show a true representation of the programme’s feasibility as people with PD can have poor memory, influencing attendance as well as possible other commitments that may impact their attendance. The small sample size in this feasibility study may also increase the chance of type 2 errors in the proposed secondary outcome measures.

In conclusion, there is a need for non-pharmacological evidence-based interventions for people with PD that addresses their problems in completing everyday activities. To the best of our knowledge, this would be the first intervention programme addressing the possible attention issue leading to their inability to complete everyday activities independently. By using self-regulation and mental imagery, we postulate that the intervention programme will assist people with PD to approach actively their challenges in a goal-directed manner. This proposed feasibility study will provide data for further research to review the programme’s effectiveness with a more rigorous research design.

## Supplementary Information

Additional file 1. SPIRIT 2013 Checklist: Recommended items to address in a clinical trial protocol and related documents.

## Data Availability

Data will be stored accordingly as required by the ethics committee and will be available from the authors on request.

## Abbreviations

PD: Parkinson’s disease
SAS: Supervisory attentional system
SReMI-PD: Self-regulation and mental imagery for Parkinson’s disease
TEA: Task, enviroment and assistance
